# Stiffness analysis of 3D spheroids using microtweezers

**DOI:** 10.1371/journal.pone.0188346

**Published:** 2017-11-22

**Authors:** Devina Jaiswal, Norah Cowley, Zichao Bian, Guoan Zheng, Kevin P. Claffey, Kazunori Hoshino

**Affiliations:** 1 Department of Biomedical Engineering, University of Connecticut, Storrs, Connecticut, United States of America; 2 Department of Cell Biology, University of Connecticut Health Center, Farmington, Connecticut, United States of America; LAAS-CNRS, FRANCE

## Abstract

We describe a novel mechanical characterization method that has directly measured the stiffness of cancer spheroids for the first time to our knowledge. Stiffness is known to be a key parameter that characterizes cancerous and normal cells. Atomic force microscopy or optical tweezers have been typically used for characterization of single cells with the measurable forces ranging from sub pN to a few hundred nN, which are not suitable for measurement of larger 3D cellular structures such as spheroids, whose mechanical characteristics have not been fully studied. Here, we developed microtweezers that measure forces from sub hundred nN to mN. The wide force range was achieved by the use of replaceable cantilevers fabricated from SU8, and brass. The chopstick-like motion of the two cantilevers facilitates easy handling of samples and microscopic observation for mechanical characterization. The cantilever bending was optically tracked to find the applied force and sample stiffness. The efficacy of the method was demonstrated through stiffness measurement of agarose pillars with known concentrations. Following the initial system evaluation with agarose, two cancerous (T47D and BT474) and one normal epithelial (MCF 10A) breast cell lines were used to conduct multi-cellular spheroid measurements to find Young’s moduli of 230, 420 and 1250 Pa for BT474, T47D, and MCF 10A, respectively. The results showed that BT474 and T47D spheroids are six and three times softer than epithelial MCF10A spheroids, respectively. Our method successfully characterized samples with wide range of Young’s modulus including agarose (25–100 kPa), spheroids of cancerous and non-malignant cells (190–200 μm, 230–1250 Pa) and collagenase-treated spheroids (215 μm, 130 Pa).

## 1. Introduction

Stiffness measurement of cancer cells has been a topic of interest [1,2]. Mechanical characteristics have been considered as a biomarker to distinguish diseased tissue from healthy one [3,4]. Various conventional techniques as well as novel micromechanical devices have been used for studying mechanical properties of single cells as well as tissues. AFM [5–12], magnetic tweezers [13] and optical tweezers have been used to calculate single cell deformability [14,15] and the range of forces measured by these techniques are 5 pN-1 nN, 10^−3^ pN-10 nN and 0.1 pN-0.1 nN, respectively [16].

Atomic force microscopy (AFM) has been used for nanomechanical characterization of single cells such as blood cells [17], fibroblast [18], neural cells [19] and bacteria [12]. For cancer cell studies, Young’s moduli of ~300Pa and ~500 Pa for cancerous (MCF7) and normal (MCF10A) single breast cells, respectively, were reported based on AFM stiffness measurements [7]. AFM analysis of benign prostate hyperplasia (BPH) cells and two prostate cancer cell lines (LNCaP clone FGC and PC-3) showed Young’s moduli of ~2800 Pa, ~ 290 Pa and ~1400 Pa respectively [11]. Micromachined tools based on piezo-resistive [20], piezo-electric [21] or optical cantilevers [22] have been used to measure the nN-pN force exerted on cells. Microfabricated grippers and nanotweezers have been used for single cells or DNA, with the gripping force range of 50 μN and a gripping force resolution of 19 nN [23–25]. Another approach is the use of microfluidic devices to evaluate mechanical characteristics of cells [26–30]. Whole cell stiffness measurements were made by monitoring interaction of cells with microchannels or micro-pillars [26,27] Microfluidic channel based screening has been used to assess the relationship between stiffness of cancer cells and malignancy [28–30].

Mechanical characterization of tissue has also been an important topic. Indentation based technique [4] and ultrasound-based elastography [31] can be used for mechanical characterization of cancerous tissue. The reported Young’s modulus for normal breast tissue is 0.16–29 kPa [32] and high grade ductal carcinoma tissue from breast is ~42 kPa [4]. Most of the tissue indentation studies use samples of few centimeters or larger in size. For the study of smaller tissue samples, a micro scale indentation technique with 1–10 μN applied forces showed Young’s modulus of ~2.5kPa for up to 3 mm thick tumor from mammary glands [33]. AFM has been recently used for analysis of biopsy samples on normal and benign tissues, showing different stiffness peaks ranging in 1-10kPa [34]. Since AFM is a surface based method, it may not be useful to study three dimensionally constructed samples such as cancer spheroids.

Cancer spheroids, spherical clusters of cancer cells, have been applied as *in vitro* 3D tumor models [29,35–37]. They have been utilized to study physiological conditions such as nutrient gradients [38], or difference in proliferative cells towards the core of a 3D tumor [39], or molecular changes associated with tumor formation [40]. They have been studied as model tumors in lab-on-a-chip systems [41,42]. Optical microscopy has been used to characterize mechanical integrity. Intercellular connections were assessed by the expression levels of β1-integrin and E-cadherin through immunofluorescence [43]. Although spheroids are believed to better represent 3D and mechanical characteristics [44], mechanical stiffness of spheroids have not been characterized because they are relatively large (100–1000μm) compared to single cells (15–30 μm) and smaller than tissue sections. Although in vitro 3D models in such size ranges, including tumor spheroids of breast [45] ovarian [46], colon [47], bladder [48], prostate [49], and cervical [50] cancer, and normal tissue organoids [51,52] have been of increasing interest, the tools conventionally used to study single cells or tissue samples do not match the force ranges needed in the analysis of multicellular models sized from 100μm to 1mm.

In this paper, we describe a new method to measure the stiffness of 3D spheroids. We have developed force-sensing micro tweezers designed and fabricated for spheroid stiffness measurement. With one of the cantilevers displaced by a piezo-bimorph actuator, the tweezers work like a pair of chopsticks to compress a cancer spheroid. Different from the previous studies, our tweezers comprise replaceable force-sensing cantilevers that can be easily changed to work with different forces ranging from less than one hundred nN to one mN. The bending of cantilevers is optically tracked using pattern matching technique to find the force exerted on the cantilevers. The setup was first tested using agarose with known stiffness ranges [53,54], which was followed by successful measurement of spheroids prepared from different types of cancerous and normal breast cells. Stiffness analysis will provide a new tool to assess mechanical characteristics of in vitro 3D models. In order to demonstrate feasibility of further studies in drug studies, we conducted measurements with collagenase treated spheroids.

## 2. Material and method

### 2.1 Fabrication

**Fig 1:**
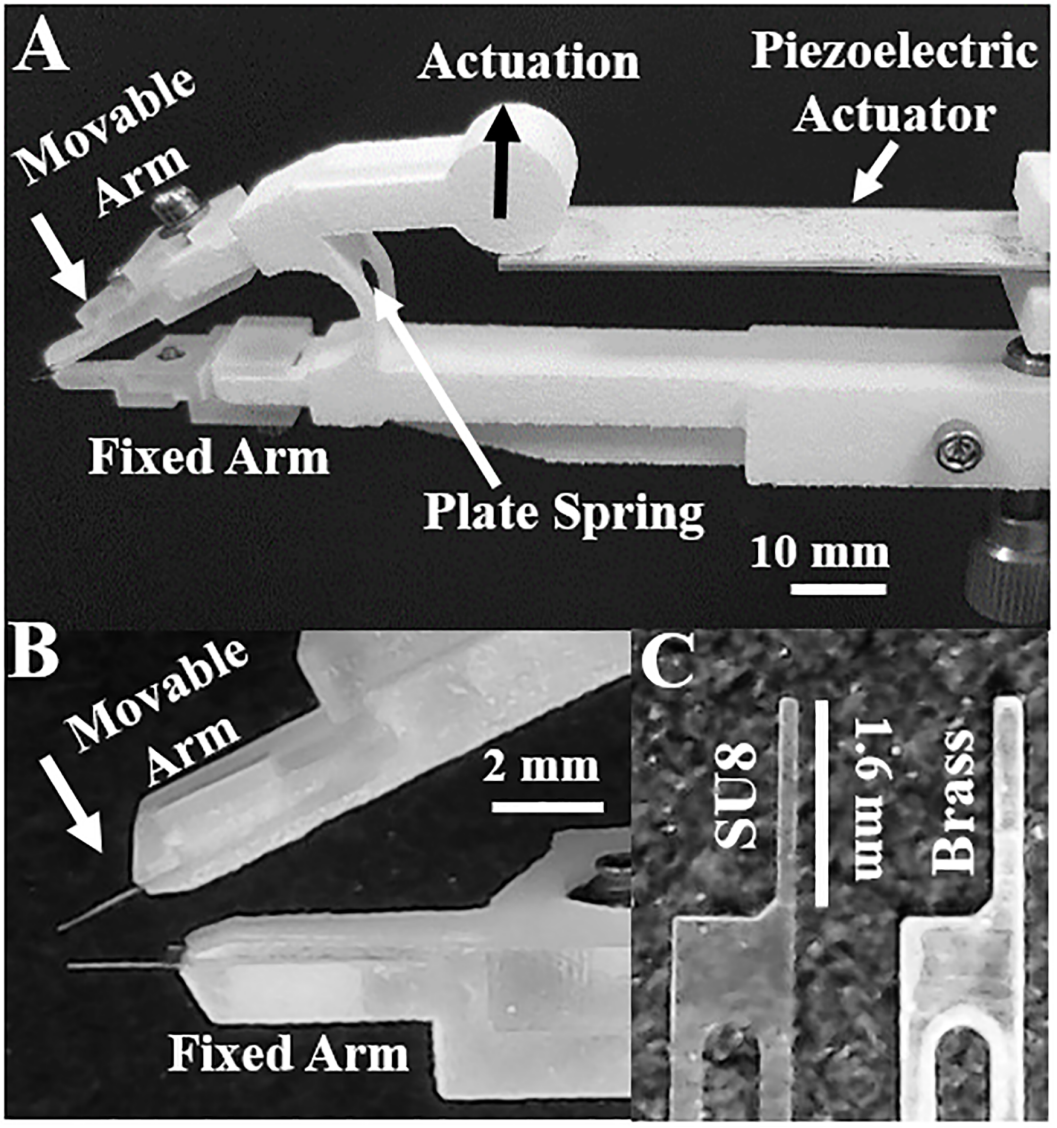
A. Microtweezer system showing the three main components: tweezer arms with a flexible plate spring, a bimorph piezo actuator and two cantilevers as the force sensing tips. The moving arm was actuated (direction indicated by the arrow) to move the cantilever closer to the fixed arm. B. The cantilever holder along with SU8 micro-cantilevers. C. Micro-cantilevers were made from SU8 and brass to obtain a wide measureable force range of sub hundred nN to more than 1 mN.

The microtweezer system consists of three components: tweezer arms with a flexible plate spring, a bimorph piezo actuator and two cantilevers as the force sensing tips. The tweezer arms comprise a fixed and a moving arm which are connected by a plate spring (Fig 1A). The tweezer arms and 0.8 mm thick flexible spring were designed using SolidWorks and printed as a single part through selective laser sintering (SLS) using nylon powder (Shapeways, NY). This design allows the use of a single bimorph piezoelectric actuator (Steminc, FL), which actuates the moving arm (Fig 1A, direction indicated by black arrow), to produce controlled displacement of the arm. Cantilever holders were 3D printed through stereolithography of UV curable acrylic polymer (Shapeways, NY) to fit at the end of the arms. The cantilever holder (Fig 1B) facilitates installation of replaceable micro-cantilevers of different materials useful, for adjusting force range.

Two types of force sensing cantilevers have been fabricated from SU8 (2.2 GPa) and brass (100 GPa) through different fabrication processes (Fig 1C). SU8 micro-cantilevers were fabricated using photolithography. The polymer film (thickness: 15 μm) was spin coated on aluminum foil taped on a glass slide. After UV exposure and development, cantilevers on the aluminum film were exposed to acetone to facilitate release from the aluminum substrate. The brass cantilevers were fabricated through wet etching. A 25 μm-thick brass film (McMaster-Carr, IL) was sandwiched between negative photoresist films (Micromark, NJ). After standard UV exposure and development, brass was subjected to wet etching with ferric chloride solution (Micromark, NJ) at room temperature for 30 mins. The patterned photoresist films were removed using acetone to recover the brass micro-cantilevers. The tip dimensions and their spring constants are compared in Table 1. Micro-cantilevers of same material were installed into the tip holders of moving and fixed tweezer arms.

**Table 1:** Two materials were used to make replaceable cantilever tips. The stiffness and force range for each cantilever is indicated.

Material	*L* (μm)	*W* (μm)	*T* (μm)	*E* (GPa)	Spring constant (N/m)	Typical force range (μN)
**SU8**	1600	100	15	2.2	(5.3 ± 0.3) ×10^−2^	0.053–5.3
**Brass**	1600	150	25	100	(1.34 ± 0.09) ×10	13–1300

### 2.2 Principle of force sensing

**Fig 2:**
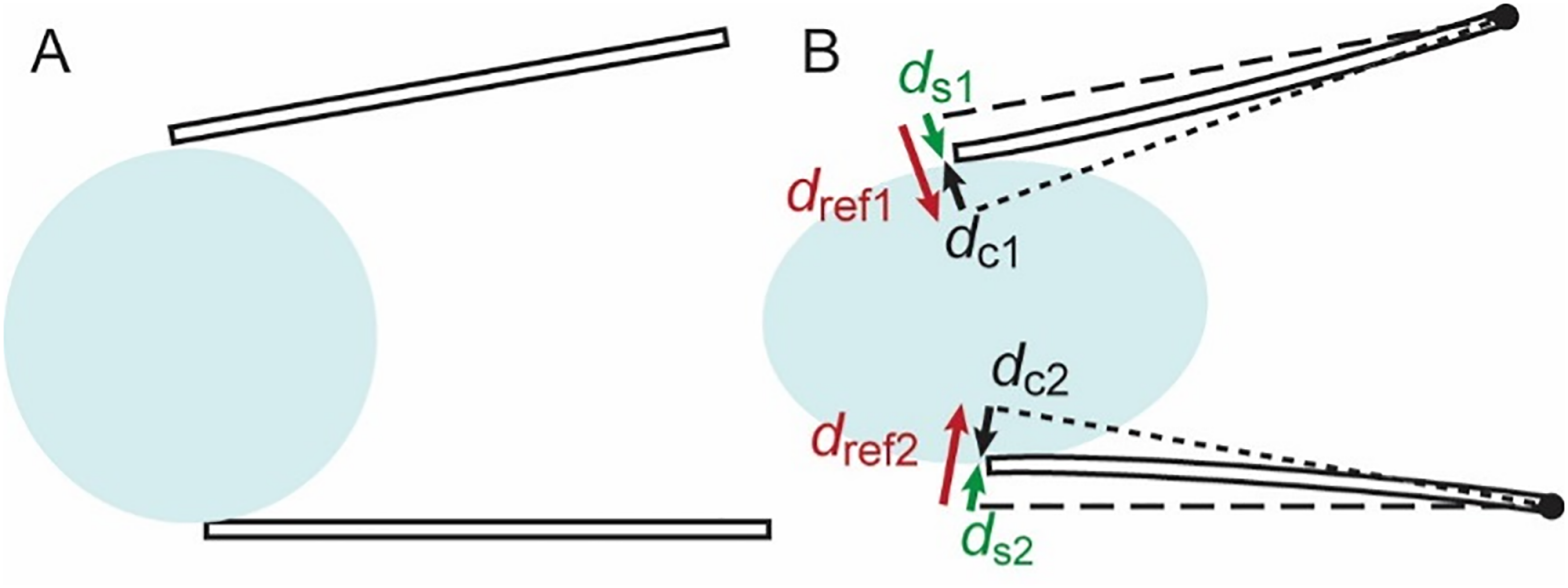
Schematic showing the principle of force and stiffness measurement. The sample was placed between the cantilevers (A) and stepwise motion of the moving arm produced bending of the flexible cantilevers (*d*_c_) and sample indentation (*d*_s_). The reference cantilever deflection (*d*_ref_) is found in the reference measurement where there was no sample between the cantilevers.

Fig 2 illustrates the principle of stiffness measurement using the dual cantilever system. The sample is placed between the two cantilevers (Fig 2A). When the cantilevers compress the sample, a force perpendicular to the cantilever tip is exerted on the sample. The dashed lines in Fig 2B show the original positions of the cantilevers as shown in Fig 2A. When the cantilevers are actuated without the sample, they will come in the positions shown with the dotted lines. The tip-tip distance between the dotted and dashed lines gives reference deflection *d*_ref_ for each cantilever. When there is a sample, the cantilevers indent the sample by distance *d*_s_ and are bent due to the stiffness of the sample. The cantilever bending *d*_c_ can be found from the following relationship:

$$d_{ref}=d_{c}+d_{s}$$ (1)

The force applied by a cantilever to the sample and the force applied back from the sample to the same cantilever are equal and opposite to each other.

$$\begin{array}{c} \{\begin{array}{c} F_{\text{c}1}=k_{\text{cantilever}}\cdot d_{\text{c}1}=k_{\text{sample}}\cdot d_{\text{s}1}\\ F_{\text{c}2}=k_{\text{cantilever}}\cdot d_{\text{c}2}=k_{\text{sample}}\cdot d_{\text{s}2} \end{array} \end{array}$$ (2)

where *k*_cantilever_ and *k*_sample_ are the spring constants of the cantilever and sample, respectively, in the direction of applied force.

The force is found through analysis of beam bending [55,56]. When a bending force *F*_c_ is applied at the end of a cantilever (width *w*, thickness *t* and length *L*), the cantilever experiences maximum deflection at the end:

$$y_{max}=\frac{L^{3}}{3EI}.F_{c}$$ (3)

Where *E* is young’s modulus of the cantilever material, and *I* is second momentum of area given by:

$$I=\frac{1}{12}wt^{3}$$ (4)

Thus, the spring constant of the cantilever is:

$$k_{cantilever}=\frac{3EI}{L^{3}}$$ (5)

The values of *d*_ref_, *d*_c_, and *d*_s_ are found from the optical analysis described later. Plugging *k*_cantilever_ and *d*_c_ into Eq (2), we find the force (*F*_c_) experienced by the spheroid. Once *F*_c_ is found, the spheroid spring constant (*k*_spheroid_) is found from the spheroid indentation (*d*_s_) and Eq (2).

### 2.3 Microtweezer characterization using agarose

**Fig 3:**
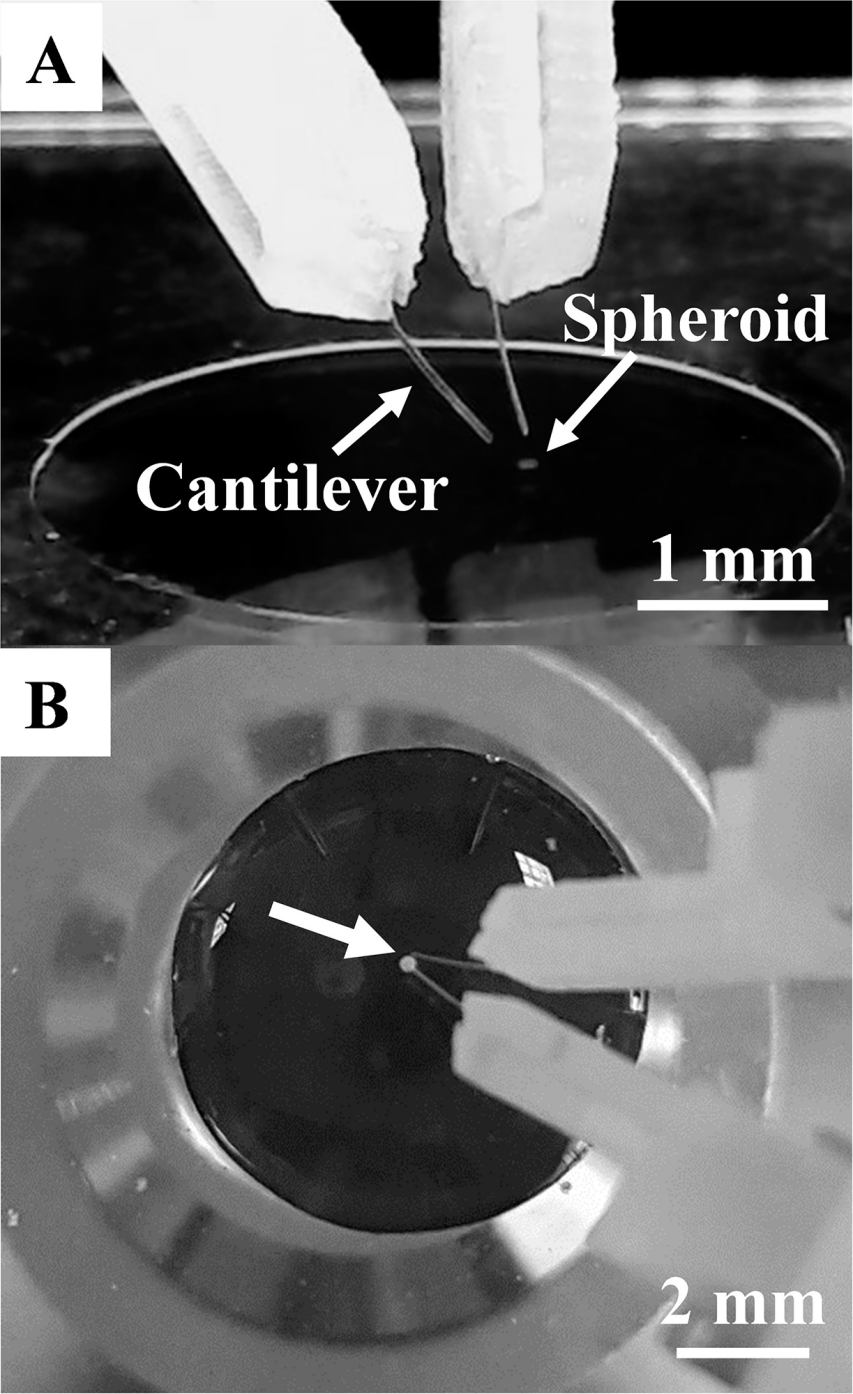
(A) The experimental set up with a well containing a spheroid. B. The cantilever tips were immersed into the media to access the spheroid (arrow) to apply step compression.

The device was first tested using agarose (SeaKem ME agarose) pillars cured at room temperature with known concentrations. Agarose pillars of 2.8, 2.4. 2.0, 1.6 and 1.2% were made (n = 3 samples for each concentration). They were punched out from an agarose sheet (250 μm thick) using a 250 μm diameter punch. A sample well (Diameter: 5mm, Height: 130 μm) was made on a glass coverslip and the pillars were suspended in the well that contained 50 μl water to prevent drying of agarose during the experiment. Brass micro-cantilevers (L: 1.6 mm, w: 150 μm, t: 25 μm) were used for this experiment. Voltage input of 1.1 V/step from 0 V to typically ~40V (maximum 96 V) was given to the piezo actuator, which produced an average displacement of 4.9± 0.5 μm/step at the tweezer tip. The samples were imaged with a 10× objective placed below the sample (Fig 3). For each measurement, a control experiment, where tweezers are operated without the sample, was conducted as a reference. Optical images of each compression stage were captured for further analysis and stiffness calculation.

### 2.4 Stiffness analysis using pattern matching

**Fig 4:**
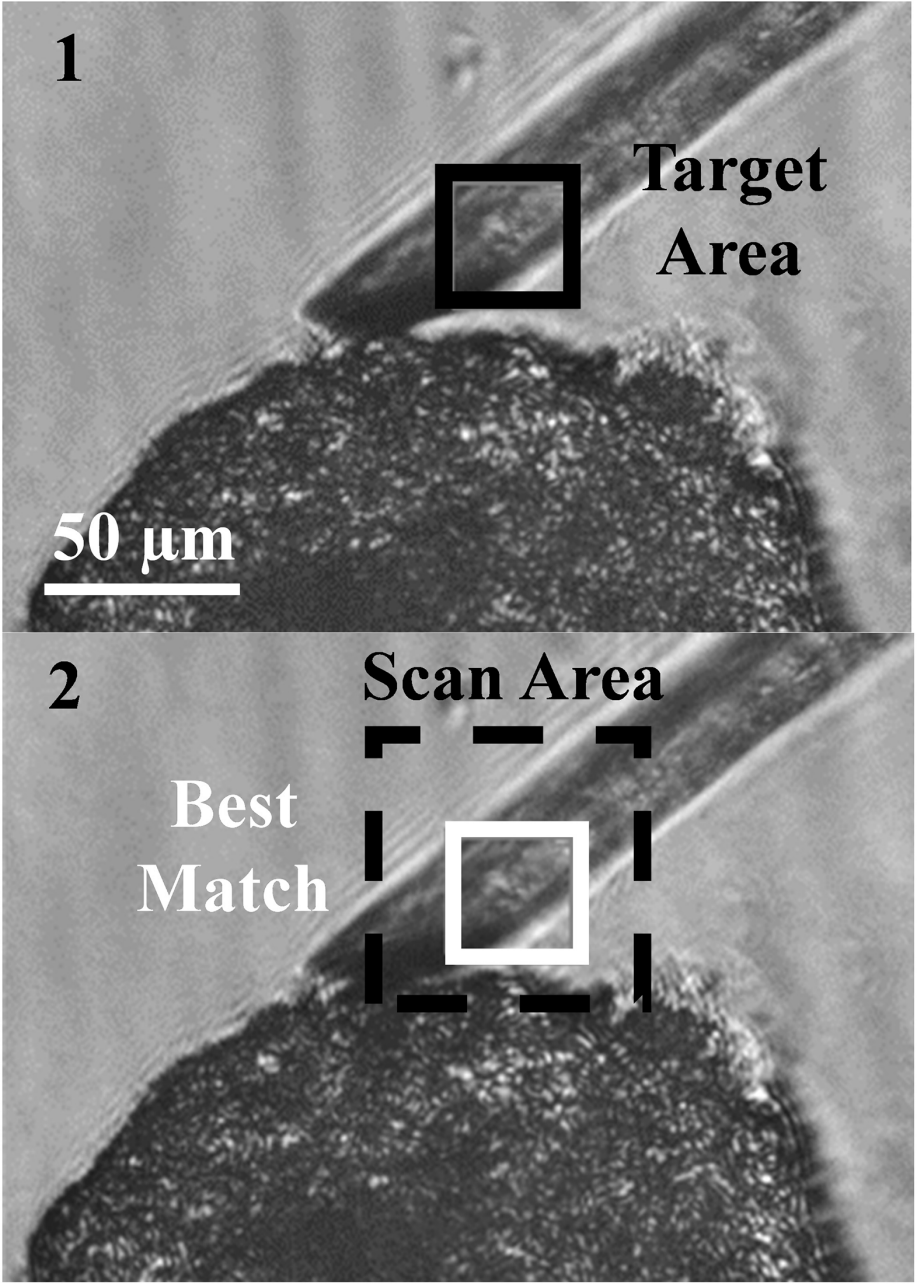
The pattern matching algorithm written in MATLAB marked a target area (black square, 50×50 pixels) in image 1 and searched a larger scan area (dashed black square, 110×110 pixels) for a match of the target area in image 2. The best matched area (2, white square) will be used as the new target in the next step.

A custom built MATLAB program was used to analyze optical images for each compression step. In the MATLAB program, we used vector dot product for pattern matching. When N images were captured for each compression, N positions of cantilever tips were found for each experiment. An image tile (50×50 pixels) containing the edge of a cantilever tip was chosen from the 1^st^ image (Fig 4, panel1) and was defined as the 1^st^ target area. The scan area (110×110 pixels) in the second image that surrounds the location of the 1^st^ target was searched with the matching algorithm to find the best matched area (50×50 pixels) as shown in Fig 4, Panel 2. The dot product of the normalized target vector (50×50 = 2500 elements) and a normalized subset vector (50×50 = 2500 elements) of the scan area is calculated as the subset area sweeps through the scan area. The subset vector that gives maximum dot product with the target vector defines the best matched area in the 2^nd^ image. Now this area is defined as the 2^nd^ target image and best match is found in the 3^rd^ image. This process is repeated for all N images, i.e. the target in the (i-1)^th^ image is used to find the match in the i^th^ image until i reaches N. The position of each tip is tracked and stored, and the distance between the two tips is used to calculate the tweezer tip deflection. The same analysis is run for a control experiment, where no sample is placed between the tips. The data is used to calculate sample deflection and force exerted on the sample as discussed with Fig 2.

**Fig 5:**
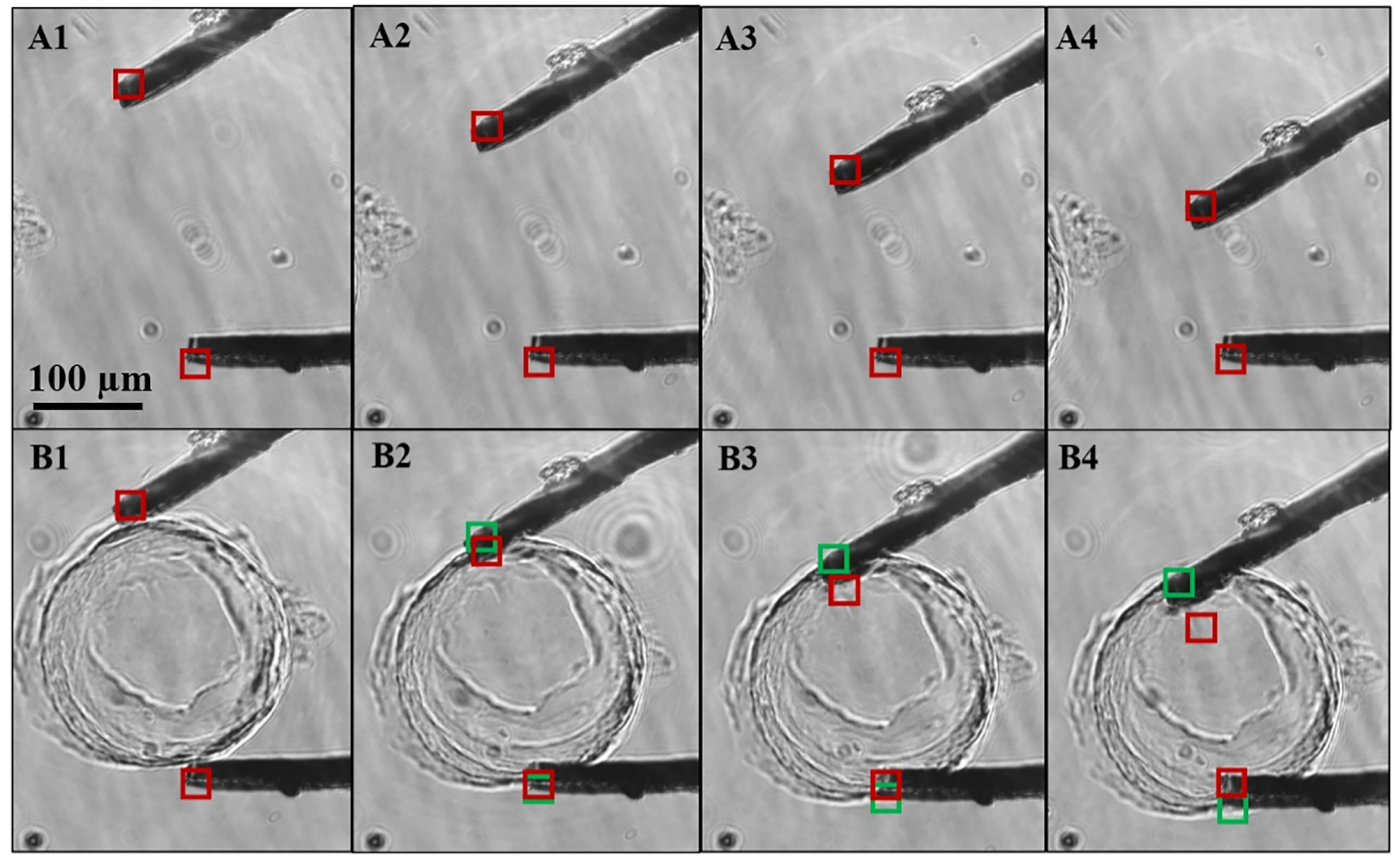
The microtweezer system was verified through measurement with agarose pillars (250 μm diameter) with different concentrations. Panels A1-A4 shows the reference measurement where there is no sample between the cantilevers. Panels B1-B4 are the corresponding images with a sample. Panel 1–4 represents step 0, 6, 12 and 18, respectively. The red image tiles (red square) were selected and tracked from the reference images while the green image tiles were tracked from sample images. The shift in the position of the red and green tile with step compression of the agarose pillar shows the bending of cantilevers and sample indentation. S1 Movie is available.

**Fig 6:**
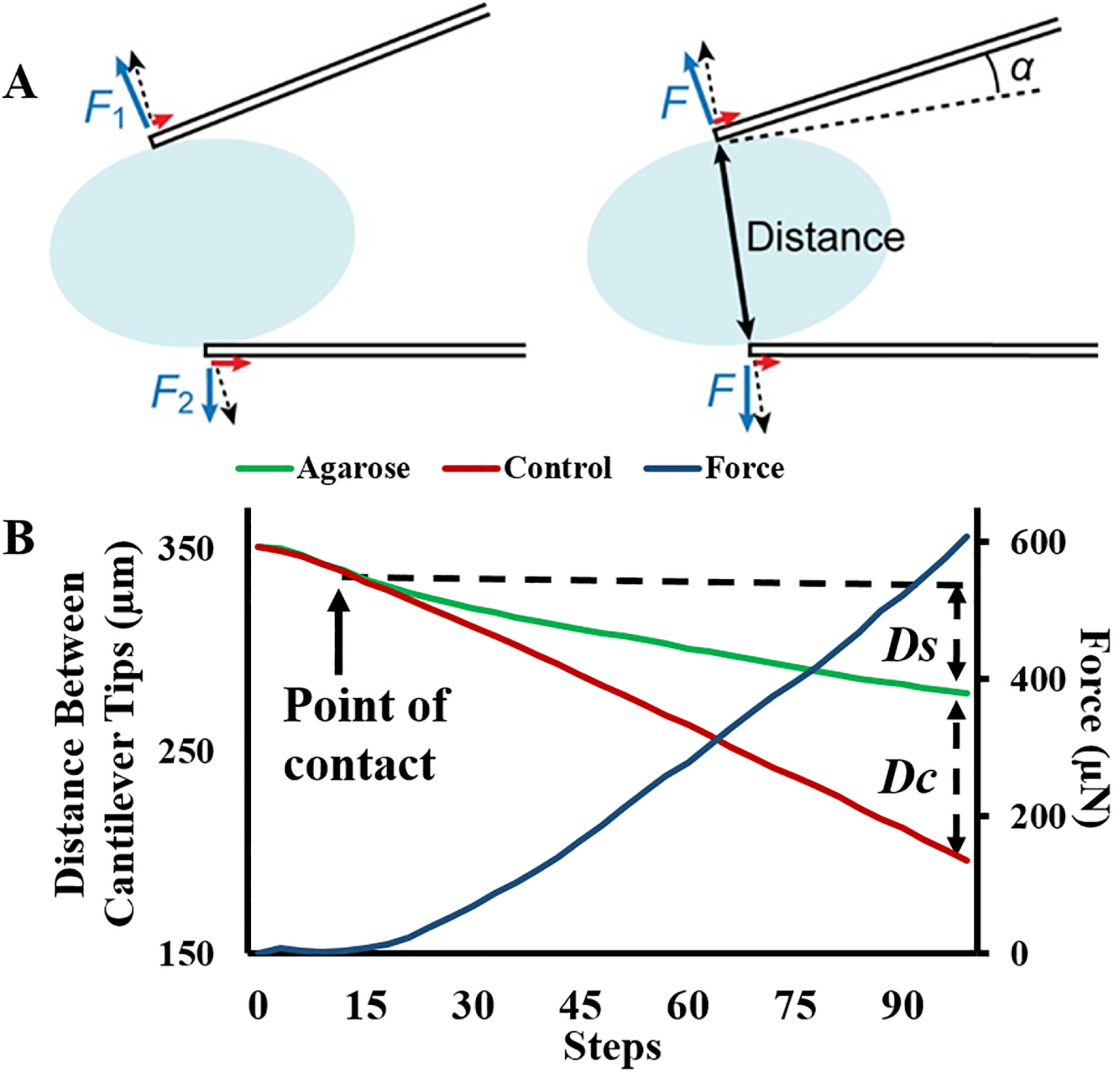
A. The force diagram of the system. B. The sample indentation and cantilever bending were calculated from the distance between the cantilever tips. The two plots start from the same point and track the same trajectory until the cantilevers come in contact with the sample (arrow, point of contact) thereby showing a deflection in sample plot (green). Secondary axis: The displacement values and the cantilever spring constant are used to calculate the force exerted by the cantilevers on the sample.

Fig 5 shows the compression of agarose pillars (Fig 5B1–5B4) and the corresponding control measurements (Fig 5A1–5A4, S1 Movie). The panels show compression at 0, 10, 20 and 30 steps (1.1 V/step) representing the total 147 μm displacement of moving cantilever. The green box depicts the position of the cantilever tip while compressing the sample and red box depicts the position of the tip when there is no sample between the cantilevers. Fig 6A left shows the force diagram of this experiment. Even when the two cantilevers are identical, the bending of a cantilever may be different to that of the other cantilever, because the force component parallel to its respective cantilever (red arrows in Fig 6A) may be different.

From Eq (2)

$${(F}_{c1}+F_{c2})/2=k_{cantilever}\cdot (d_{c1}+d_{c2})/2=k_{sample}\cdot (d_{s1}+d_{s2})/2$$ (6)

Relationship between *k*_cantilever_ and *k*_sample_ can be evaluated by the averages of the forces and distances. Here we calculate average forces with respect to the tip-tip distance *D* (Fig 6A right). In other words, we consider the two cantilevers as a single spring whose deformation is defined simply by the distance between the tips, cancelling the asymmetry.

Fig 6B shows the distance between the cantilever tips plotted at each compression step. The green plot shows the distance between the cantilever tips when there is a sample. The deflection of the cantilever and sample can be calculated from this measurement. Components of distance changes due to cantilever actuation (*D*_ref_), sample indentation (*D*_s_), and cantilever bending (*D*_c_) satisfy the following relationship:

$$D_{ref}=D_{s}+D_{c}$$ (7)

The deflection normal to the cantilever has an angle in the direction of the distance measurement (Fig 6A right). The cantilever deflection is found by:

$$d_{c1}+d_{c2}=D_{c}/cos\;\alpha$$ (8)

In this case, the angle was α = 15° for closed and 12° for open cantilevers. The normal cantilever deflection values were calculated using an average of cos12° = 0.978 and cos15° = 0.966.

The force *F*_c_ exerted by the cantilever on the sample was calculated using *d*_c_ = (*d*_c1_ + *d*_c2_) /2, considering the average force of the two cantilevers (Fig 6B). From Eqs (2) and (8), the force *F*_c_ is found as

$${F_{c}=(k}_{cantilever}/(2\text{cos}\;\alpha ))\cdot D_{c}{=k}_{t}\cdot D_{c},$$ (9)

where we define *k*_t_ = (*k*_cantilever_ / (2 cos α)) as the stiffness of the pair of tweezers.

### 2.5 Cantilever calibration

Table 1 summarizes characteristics of cantilevers we used. The typical force ranges were calculated using the spring constant, the estimated optical resolution of ~1μm and the maximum cantilever bending of ~100 μm we have observed in our experiment. The spring constants shown are averages of 6 probes for each material. The spring constant of a brass cantilever was measured using an analytical balance (20g × 0.001g) and a precision mechanical stage. The mechanical stage moves the cantilever tip with incremental steps of 30 μm under microscopic observation, allowing it to push an edge of a glass coverslip placed on the balance. The force acting on the cantilever was directly measured from the balance. Forces from 0 N to ~ 3.5mN, corresponding to zero to a few hundred mgf (milligram force), were applied typically in 8–9 steps, and a linear fit to the force-displacement relationship provided the spring constant (*R*^2^ > 0.998). The SU8 cantilevers were measured in two steps. First, a reference cantilever (*L* × *W* × *T* = 9 mm × 0.6 mm × 25 μm) was manually cut from a 25 μm-thick brass film (McMaster-Carr, IL) using a scalpel. This cantilever has a spring constant comparable to the SU8 tips, but can be applied with much larger forces to be calibrated with the analytical balance. Forces from 0 μN to ~600 μN were applied in 10 steps and the spring constant of 2.53 ×10^−1^ N/m was found as a linear fit (*R*^2^ > 0.98). Second, an SU8 cantilever was pushed against the reference cantilever to find the relative stiffness. The two cantilevers are aligned in a tip-to-tip contact and was displaced by a precision mechanical stage to bend each other. Displacement of the two tips was optically tracked to find the spring constant of the SU8 lever relative to that of the reference lever. The same MATLAB program used for spheroid indentation measurements was used for this analysis.

### 2.6 Young’s modulus estimation using a finite element analysis (FEM)

The Young’s moduli of the agarose pillars were found using the finite element analysis software COMSOL (version 5.2)-solid mechanics (stationary) module, where each pillar was modeled as a cylinder (height: 250 μm, radius: 125 μm). A user defined material was assigned to the sample whose Young’s modulus could be adjusted to match the force-indentation relationship found during microtweezer experiment. The maximum compression image from the experiment was used to find the contact area between the sample and the cantilever in the COMSOL simulation, where the cantilever width and the length measured from the image were used to define the contact area. Two rectangular contact areas (width: 150 μm, length measured from the image) were defined along the walls of the sample to apply the force exerted by the cantilever tips on the sample. The model was meshed using normal free tetrahedron. Sample indentation (*d*_s_) and force (*F*_c_) exerted by the cantilever tips on the sample, obtained from optical pattern matching, were used to estimate the effective Young’s modulus that gave closest match to the measured indentation *d*_s_ at the applied force *F*_c_.

### 2.7 Mechanical characterization of agarose using microindentation

**Fig 7:**
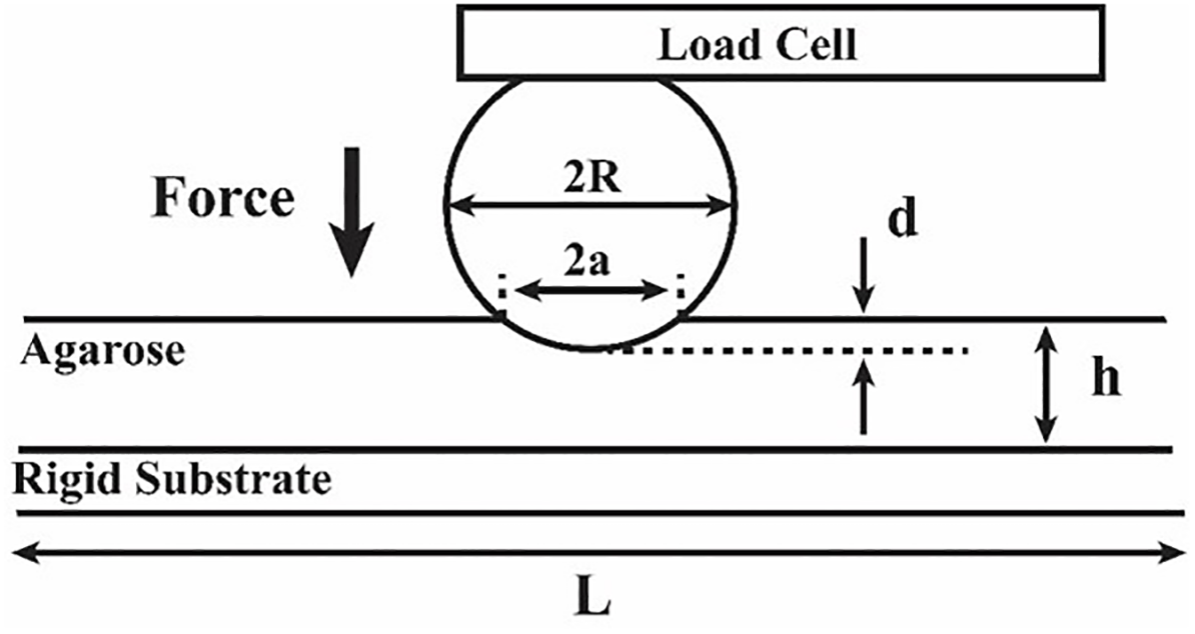
Microindentation set up for mechanical characterization of agarose using stainless steel sphere and 100g load cell.

Mechanical characterization of agarose was also conducted using the standard microindentation technique [57]. The same agarose concentrations (1.2, 1.6, 2.0, 2.4 and 2.8% (wt/v)) as tested with the microtweezers were used to compare the young’s moduli. The experimental set up (Fig 7) consisted of a stainless steel sphere (diameter: 6mm) which was attached at the end of a 100 g load cell. Agarose (25×25×6 mm) was placed on a stationary rigid platform under the sphere. The microindentation arm moved 0.021mm for every step. A side view optical image was captured for each step of compression and analyzed using Image J to calculate the contact radius (*a*). The reading of load for every compression step was recorded and used to calculate force (*F*) exerted on the agarose sample. When the indentation depth (*d*) and contact radius (*a*) are small compared with the agarose thickness (*h*) and length (*L*), respectively, to meet *d*/*h* < 10% and *L*/*a* > 12 [58], the young’s modulus was given in the following equation [59]:

$$E=\frac{(1-\nu^{2})3RF}{4a^{3}}$$ (10)

where *R* is the radius of the sphere, *ν* (= 0.5) is the Poisson’s ratio [57] and *E* is the Young’s modulus of the sample. With our samples, *L* and *h* were large enough to satisfy *d/h* < 3% and *L/a*> 23.

### 2.8 Spheroid culture and stiffness analysis

**Fig 8:**
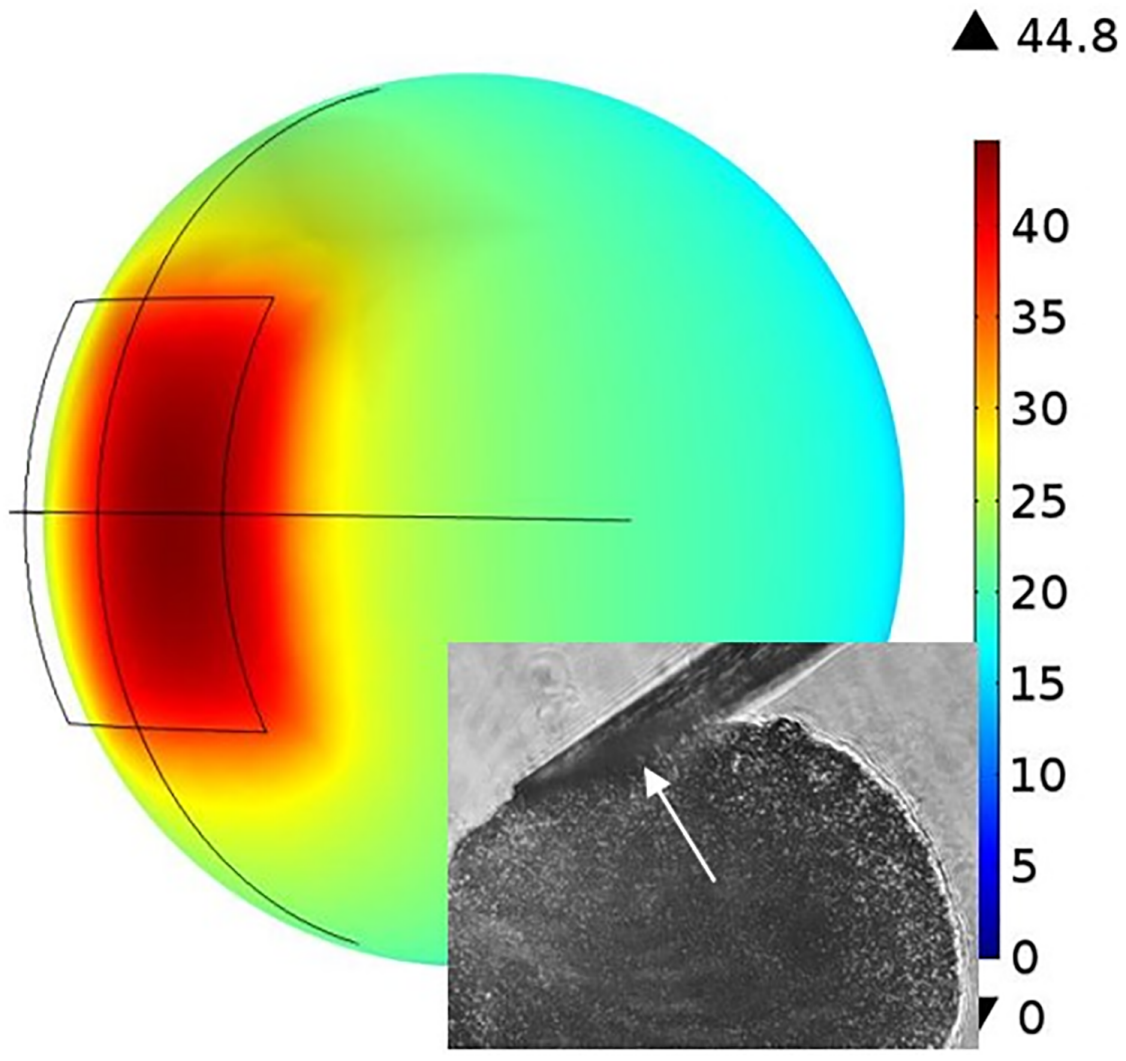
COMSOL model of spheroid for calculation of Young’s modulus. The rectangular area shows the area compressed by the cantilever. The color bar represents total displacement in microns. Inset shows the area of spheroid covered by cantilever at maximum force application.

Two cancerous breast cell lines (BT474, T47D) and a normal epithelial breast cell line (MCF10A) were used to grow spheroids (American Type Culture Collection, Manassas, VA). BT474 and T47D were cultured in DMEM, 10% FBS and 1% Pen/Strep (Life Technologies). MCF10A was cultured in MEGM (Lonza, MEGM Bullet kit). A 96 well, U bottom, plate (Thermo Fisher Scientific) was coated with 1.6% agarose and sterilized under UV for one hour. Cells in suspension were added to each well and incubated. The number of cells seeded in each well was chosen for each cell line to obtain spheroids ranging from 180–200 μm. T47D was seeded with 900 cells/ well, MCF10A and BT474 were seeded with 1000 cells/well. An average diameter of 190±11, 200±12 and 200±17 μm was measured using ImageJ for BT474, T47D and MCF10A spheroids, respectively. Spheroids (n = 6 per cell line) formed were used for stiffness analysis at day 5. During this experiment, SU8 cantilevers (L: 1600 μm, w: 100 μm, t: 15 μm) were used. The spheroid deflection values were calculated with the protocol used for agarose pillars. Young’s modulus was estimated through a COMSOL simulation where the spheroid was modeled as a sphere (Fig 8). Two rectangular areas were made on the sphere for force application. Length and width of the rectangle correspond to the actual contact length measured from the image (Fig 8, inset) and the width (100 μm) of the cantilever, respectively. The measured contact area and spheroid diameter of each sample were used to build a spherical model in the COMSOL simulation.

### 2.9 Collagenase treatment

Spheroids made from BT474 harvested at day 5 were treated with 0.1% collagenase solution made in serum free DMEM for three hours to degrade collagen and were washed three times with PBS in a centrifuge at 1000 rpm for 2 mins. The stiffness experiment was conducted for n = 6 samples.

### 2.10 Viscoelasticity

**Fig 9:**
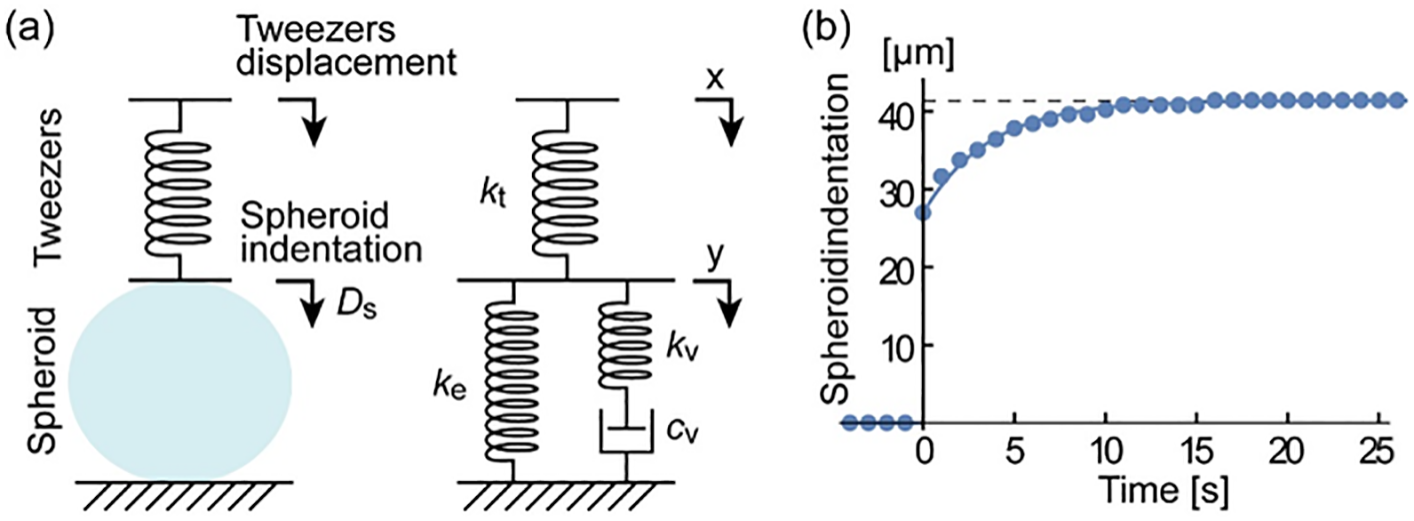
(a) Standard linear solid model applied to the spheroid in our system. (b) Measured response of a BT474 spheroid to a step input, where the tweezers closed rapidly in a single step.

The shape of a cell is defined by the tension of the elastic membrane that contains the intracellular fluid within the cell [60]. Because of this structure, cells demonstrate viscoelasticity, which is a combined characteristic of viscous and elastic materials. Here we apply a standard linear model [61,62] as illustrated in Fig 9(a). In this model, the spheroid consists of two components in parallel: the elastic spring (*k*_e_) and the Maxwell arm, which includes a spring (*k*_v_) and a dashpot (*c*_v_) in series. The tweezers are modeled as a simple spring (*k*_t_) as given in Eq (9).

The output *y*(*t*) to a unit step input *x*(*t*) of this system is analytically found as

$$y\left(t\right)=\{\begin{array}{ll} 0 & \left(t<0\right)\\ \frac{k_{\text{t}}}{k_{\text{t}}+k_{\text{e}}}+\left(\frac{k_{\text{t}}}{k_{\text{t}}+k_{\text{e}}+k_{\text{v}}}-\frac{k_{\text{t}}}{k_{\text{t}}+k_{\text{e}}}\right)\cdot \text{exp}\left(-\frac{t}{\tau }\right)\; & \left(t\geq 0\right) \end{array}\;\text{where}\;\tau =\left(\frac{1}{k_{\text{t}}+k_{\text{e}}}+\frac{1}{k_{\text{v}}}\right)\cdot c$$ (11)

When a rapid indentation is given, the spheroid acts as two springs (*k*_e_ + *k*_v_) connected in parallel. After creeping deformation in the viscous components, only the elastic component *k*_e_ is effective. Dots in Fig 9(b) show the measured step response of BT474 spheroids. From the curve fit, the time constant for the exponential creeping was found to be *τ* = 3.6 s. All compression experiments, other than this step analysis, were conducted in a ‘slow’ indentation process, where a series of small steps were applied with time intervals of 1s and the entire indentation takes ~30s. At this rate, the contribution from the Maxwell wing is less than 5%. When we use the elastic components *k*_e_ of a BT474 spheroid shown in Table 2 and the tweezer stiffness *k*_t_ given in Eq (9), the parameters for the Maxwell wing are given as:

$$k_{\text{v}}=1.9\times 10^{-2}\left[\text{N}/\text{m}\right],c_{\text{v}}=4.4\times 10^{-2}\left[\text{N}\cdot \text{s}/\text{m}\right]$$ (12)

**Table 2:** The Young’s modulus of spheroids of each cell line was calculated using finite element analysis software COMSOL.

Cell Lines	Size (μm)	Stiffness×10^−3^ (N/m)	Young’s modulus (Pa)
**BT474**	190±11	13±3.0	230±60
**T47D**	200±12	26±7.9	420±90*
**MCF10A**	200±17	76±22	1250±320**

The BT474 and T47D spheroids were significantly softer than MCF 10A spheroids

(** P-value < 0.005).

The two malignant cell lines shows a significantly softer BT474 spheroid compared to T47D spheroid

(* P-value < 0.05).

### 2.11 Cell viability test

Live/Dead cell viability assay (Thermo Fisher) was conducted on spheroids to test cell viability during and after compression by cantilevers. The microtweezers were attached to confocal microscope (Nikon A1R) stage for this experiment. Spheroid at day 5 was incubated with Live/Dead solution (2μM Calcein AM and 4 μM Ethedium Homodimer-1 solution in Phosphate buffer saline (PBS)) for two hours. The spheroid was placed in a well filled with Live/Dead solution. Microtweezers were used to compress the spheroid similar to the process explained in stiffness analysis section. Once compression was completed, the spheroid was released from the cantilevers and left in the Live/Dead solution for 10 mins. Image of mid-section of the spheroid was captured before compression, at maximum compression and 10 mins after release.

## 3. Result and discussion

**Fig 10:**
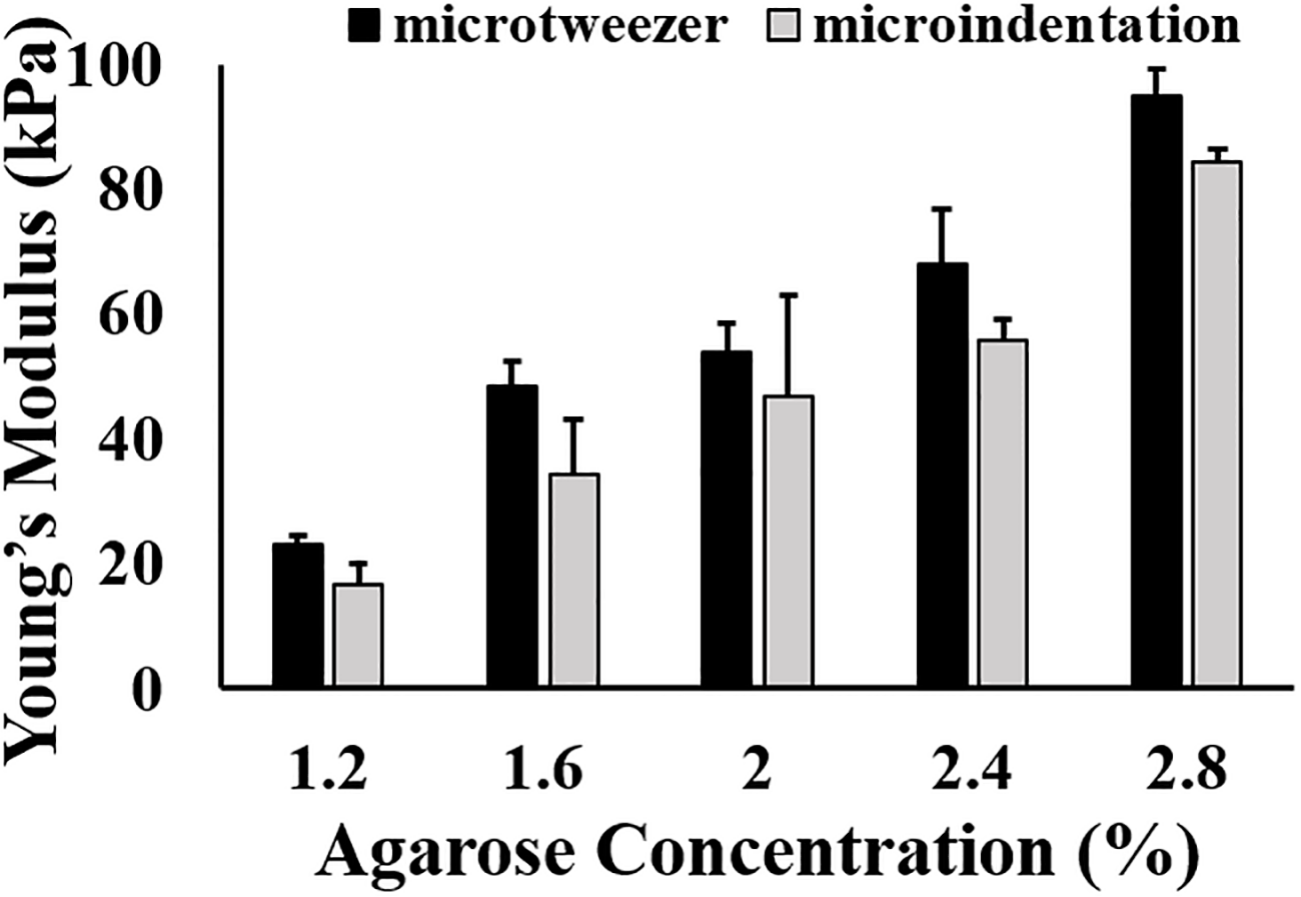
Young’s modulus of agarose measured using two techniques: Microindentation and microtweezer.

Microindentation is a well-studied method used for mechanical characterization of hydrogels and tissues in tissue engineering applications [63,64]. Fig 10 shows the Young’s modulus of agarose determined by two methods: microtweezers and microindentation. The young’s modulus obtained from the two methods are not significantly different. This comparative study indicates the efficacy of the device and method developed in this study for mechanical characterization of biological samples such as spheroids.

**Fig 11:**
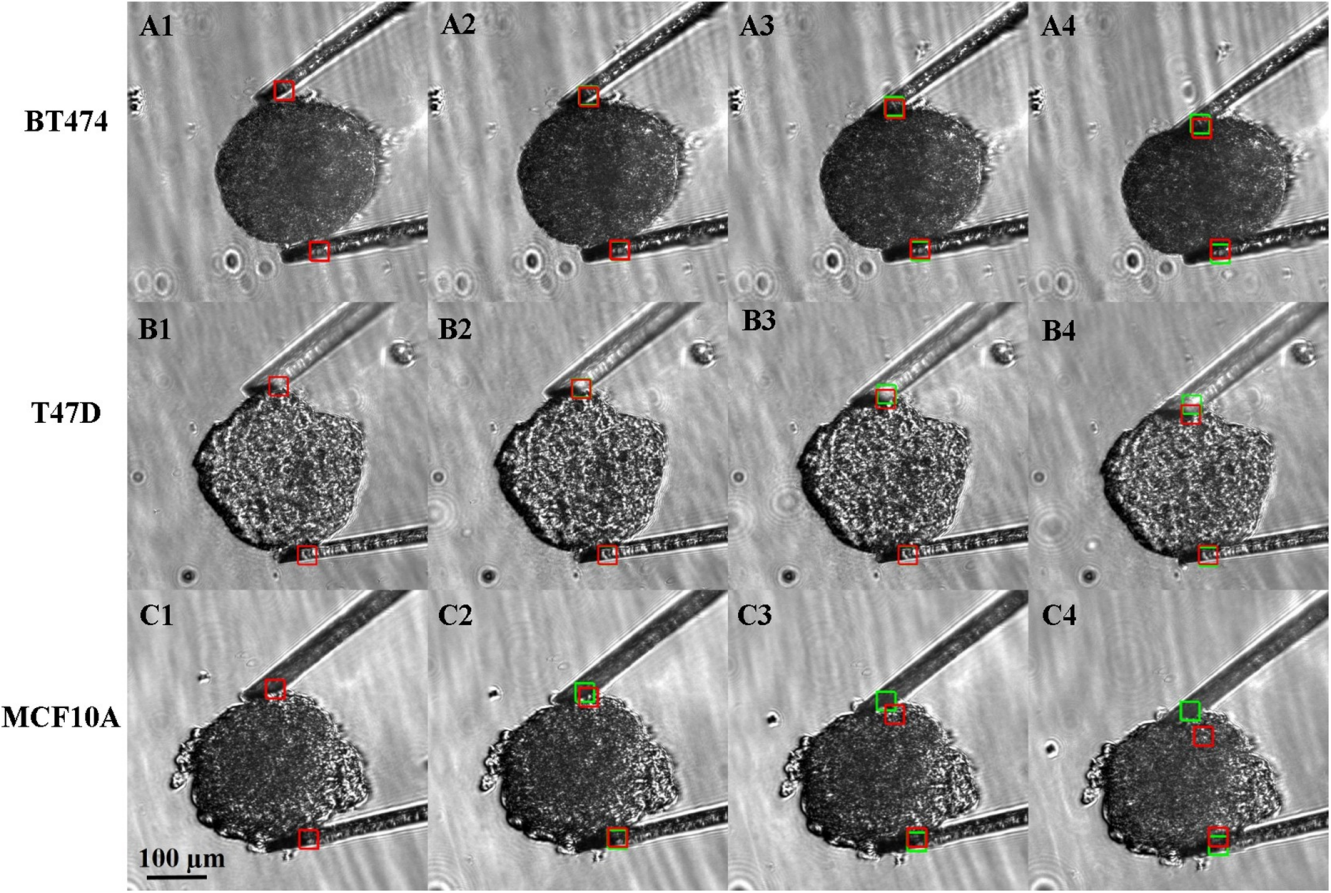
Spheroid stiffness analysis was done using SU8 cantilevers. Three cell lines BT474, T47D and MCF 10A spheroids were tested. The protocol similar to agarose pillar was applied for each cell type. Red square indicates reference and green square indicates the position of the cantilever tip with sample. The deviation in the green and red square in A4, B4, and C4 shows cantilever bending due to the spheroid. A1, B1 and C1 being the initial position for each cell line. Panel 1–4 represents step 0, 6, 12 and 18, respectively, for cantilevers. Supplementary movie clips of BT474 (S2 Movie), T47D (S3 Movie) and MCF10A (S4 Movie) compression are available.

Fig 11 shows the step compression for BT474, T47D and MCF10A (see S2, S3 and S4 Movies. The green boxes represent the bottom edges of cantilever tips tracked for each compression step. The red boxes depict the edges tracked in control experiments. As seen in Fig 11A1, 11B1 and 11C1, the outside bottom corner of the tip was centered in the target area. A step displacement of cantilever for every 1.1V supply to the moving cantilever arm was 4.3±0.9 μm. The total compression steps for each spheroid was 18. The maximum force exerted by SU8 cantilevers on BT474, T47D and MCF10A spheroids were 0.66, 1.1, 1.8 μN respectively. Cancer spheroid stiffness and Young’s modulus for three cell lines is summarized in Table 2. This study showed that malignant BT474 and T47D spheroids are approximately six and three times softer than epithelial MCF10A spheroids, respectively, with a P-value of less than 0.005. Mechanical signatures have been studied for distinguishing cancer tissue from normal epithelial tissue. Studies indicate cancer cells exhibit high deformability and lower young’s modulus compared to normal cells [65–68]. Our spheroid measurements coincides with these studies, showing lower cancer spheroid stiffness compared to normal spheroid. The Young’s modulus of spheroids is comparable with Young’s modulus of cancerous and normal cells reported in literature [7].

Extracellular matrix has been considered an important factor for drug penetration in a tumor. Treatment of spheroids with collagenase has shown better drug penetration by weakening the extracellular matrix [69]. We compared stiffness of spheroids treated/untreated with collagenase. The size and the Young’s modulus of treated samples were 215±25 μm and 130±30 Pa, respectively, while those of the untreated samples were 190±11 μm and 230±60 Pa, respectively (Table 2). These values were statistically significant with P-value of less than 0.05. There are articles that show collagen dissociation with respect to collagenase treatment [70]. Here, the change in stiffness can be attributed to digestion of collagen in the treated spheroid.

**Fig 12:**
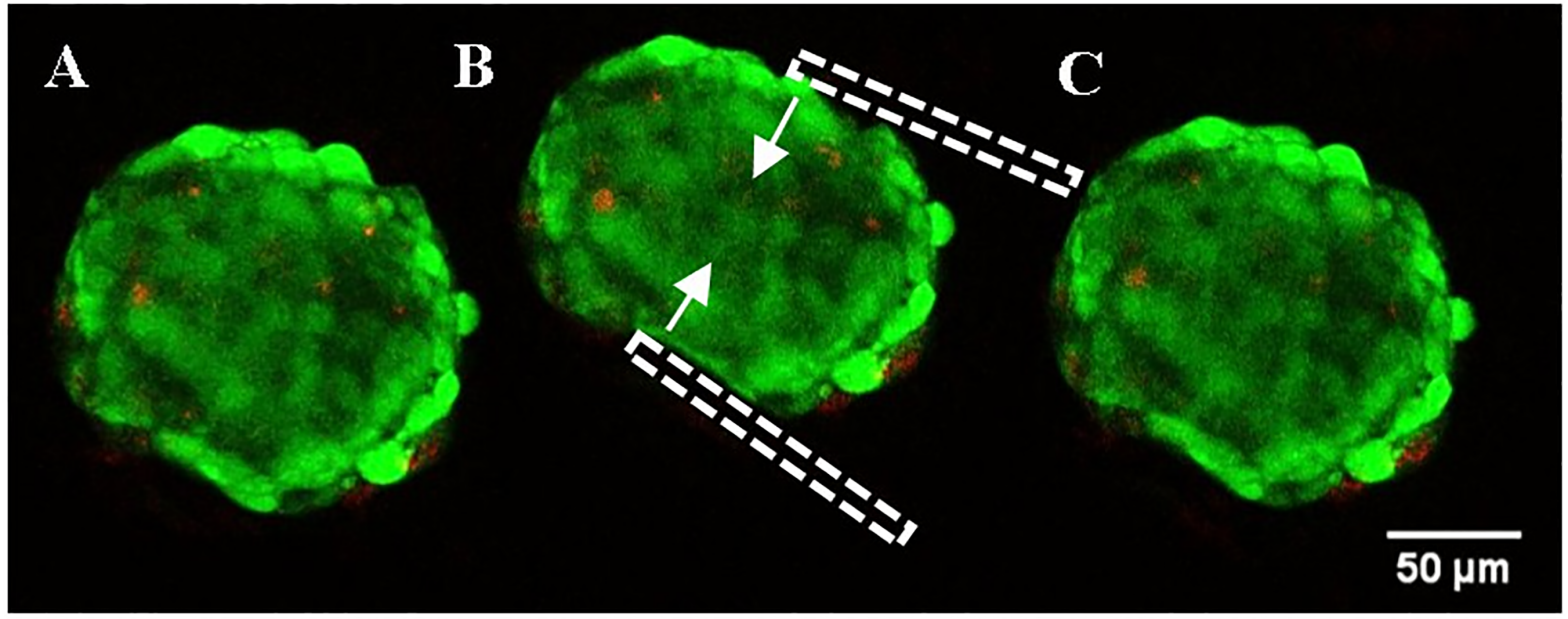
Spheroid viability test (A) before, (B) during and (C) 10 mins after microtweezer compression was conducted to show the effect of microtweezers on cell. Green shows live cells in a spheroid and red shows dead cells. There were no dead cells produced during compression (C) and after release at the point of contact with the microcantilevers (B, shown in dashed white lines) when compared to spheroid before compression (A). Thereby showing no physical damage to the sample during compression.

The cell viability results (Fig 12) showed that cells were not damaged during the microtweezing action of the cantilevers. Fig 12A shows the spheroid before compression with few dead cells (red) embedded in the spheroid. During compression (Fig 12B), the number of dead cells did not increase. The after compression image (Fig 12C) shows that the spheroid came back to its original shape and there were no dead cells around the area where the spheroid was held by the cantilevers. This shows that the device does not cause any physical damage to the cells at the point of contact and the reported Young’s modulus of spheroids is based on compression of a spheroid as a whole material.

It is also necessary to discuss time-dependent responses of materials to validate the assumption of quasi-static loading, and to further study viscoelastic characteristics. Among several models used to represent viscoelasticity, the standard linear solid (SLS) model is a simple, but useful form that well represents both instantaneous and steady-state responses. It has been adequately used in many studies of cellular [61, 62] and tissue [71, 72] viscoelasticity. The limitations of SLS include that it does not model multiple time constants induced by different factors. When components with longer time constants need to be evaluated, generalized Maxwell model [73, 74] may be applied to incorporate multiple Maxwell arms. However, our analysis with SLS is an important experimental and analytical result to discuss the time-dependent response of cancer spheroids. The model fits very well with the measured data, demonstrating the efficacy of our approach.

## 4. Conclusion

Mechanical analysis of tumor spheroids has been conducted with novel microtweezers based on two microfabricated cantilevers. The Young’s moduli of spheroids grown from malignant and non-malignant breast cell lines were measured for the first time. The image matching technique used to measure cantilever deflection permits use of cantilevers without complex integrated sensors. Forces that can be measured with replaceable cantilevers range from sub hundred nN to mN. Young’s moduli of 230, 420 and 1250 Pa were found for BT474, T47D and MCF10A spheroids, respectively. The cantilever dimensions can be easily changed to match experimental conditions or target objects. The use of our microtweezers can be further extended to the analysis of non-tumor cell aggregates including stem cell/iPSC-derived organoids [51,52,75], neuronal clusters [76,77], and 3D engineered biomaterials [51,78,79].

## Supporting information

S1 Movie Agarose pillar compression using brass cantilevers. (AVI)

S2 Movie BT474 spheroid compression using SU8 cantilevers. (AVI)

S3 Movie T47D spheroid compression using SU8 cantilevers. (AVI)

S4 Movie MCF10A spheroid compression using SU8 cantilevers. (AVI)
